# Expression of Estrogen Receptor Beta Predicts Oncologic Outcome of pT3 Upper Urinary Tract Urothelial Carcinoma Better Than Aggressive Pathological Features

**DOI:** 10.1038/srep24263

**Published:** 2016-04-07

**Authors:** Hao Lun Luo, Ming Tse Sung, Eing Mei Tsai, Chang Shen Lin, Nai Lun Lee, Yueh-Hua Chung, Po Hui Chiang

**Affiliations:** 1 Graduate Institute of Medicine, College of Medicine, Kaohsiung Medical University, Kaohsiung, Taiwan; 2 Department of Urology, Kaohsiung Chang Gung Memorial Hospital and Chang Gung University College of Medicine, Kaohsiung, Taiwan; 3 Department of Pathology, Kaohsiung Chang Gung Memorial Hospital and Chang Gung University College of Medicine, Kaohsiung, Taiwan; 4 Department of Obstetrics and Gynecology, Kaohsiung Medical University Hospital, Kaohsiung Medical University, Kaohsiung, Taiwan; 5 Center for Research Resources and Development, Kaohsiung Medical University, Kaohsiung City, Taiwan; 6 Department of Biological Sciences, National Sun Yat-sen University, Kaohsiung, Taiwan; 7 Institute of Biomedical Sciences, National Sun Yat-sen University, Kaohsiung, Taiwan

## Abstract

Upper urinary tract urothelial carcinoma (UT-UC) is rare and treatment options or prognostic markers are limited. There is increasing evidence indicating that urothelial carcinoma may be an endocrine-related cancer. The aim of this study was to analyze the prognostic effect of estrogen receptor beta (ERβ) on the outcome of UT-UC. From 2005 to 2012, this study included 105 patients with pT3 UT-UC. Perioperative factors, pathological features, and ERβ immunostaining were reviewed and prognostic effects were examined by multivariate analysis. This study divided patients into either the ERβ-high (n = 52) or ERβ-low (n = 53) group and analyzed their oncologic outcomes. All pathological features except infiltrating tumor architecture (significantly higher incidence in ERβ-low group, *p* = 0.004) are symmetric in both groups. Low ERβ expression was significantly correlated with local recurrence and distant metastasis in univariate analysis (*p* = 0.035 and 0.004, respectively) and multivariate analysis (*p* = 0.05 and 0.008, respectively). Cell line study also proved that knock down of ERβ cause less UTUC proliferation and migration. In addition, ERβ agonist also enhanced the cytotoxic and migration inhibition effect of cisplatin and ERβ antagonist cause the UTUC cell more resistant to cisplatin. This result may help identify patients in need of adjuvant therapy or develop potential targeted therapy.

Upper urinary tract urothelial carcinoma (UT-UC) is relatively rare, accounting for about 5% of all urothelial carcinomas^1^. The current standard treatment choice for locally advanced UT-UC is still radical nephroureterectomy, but the cancer-specific outcome is relatively poor. Systemic therapy might improve overall survival in patients with locally advanced disease but there is limited evidence for the benefit of neoadjuvant or adjuvant chemotherapy as most patients have renal insufficiencies and are ineligible for chemotherapy^2^. Therefore, further research to improve oncologic outcome (local recurrence and distant metastasis) is necessary. Much effort has been invested in the identification of markers to sub-classify advanced UT-UC^3^,^4^,^5^. In addition, prognostic molecular markers may also help in targeted therapy development. Previous studies have reported that urothelial carcinoma may be an endocrine-related cancer^6^,^7^,^8^. However, most studies have focused on urinary bladder urothelial carcinoma (UB-UC), not UT-UC. Hormone receptors were found to be expressed in UB-UC, and estrogen receptor beta (ERβ) was reported as a potential prognostic marker in UB-UC^8^,^9^. Therefore, the aim of this study was to identify the prognostic role of ERβ compared to other pathological factors in advanced UT-UC.

## Patients and Methods

### Study population and selection criteria

From 2005 to 2012, 188 patients with locally advanced UT-UC (pT3) underwent radical nephroureterectomy at our institution. The study excluded 73 patients with concurrent pT3 renal pelvis and ureteral urothelial carcinoma and 10 patients with tissue loss in our specimen bank. Finally, 105 patients with solitary renal pelvis or ureteral pT3 urothelial carcinoma were included in the study. The study was approved by Kaohsiung Chang Gung Medical Center Institutional Review Board (IRB number: 102–2331B) and the method we use is in accordance with the approved guidelines. Inform consents were obtained preoperatively.

### Pathological features and clinical outcome assessment

Perioperative data were obtained from patient charts. Data on commonly known prognostic pathological features, including lymphovascular invasion, carcinoma *in situ*, squamous differentiation, and tumor necrosis were recorded^10^. The follow-up protocol at our institution is postoperative fiber-cystoscopy and renal echo every 3 months during the first 2 years, every 6 months during the third to fifth year, and every year thereafter during the follow-up period. Abdominal computed tomography was performed annually to assess local or regional recurrence of the tumor and lymph node status. Local recurrence is defined as disease occurrence in the ipsilateral retroperitoneal space, and distant metastasis is defined as disease occurrence outside the residual urinary tract system and ipsilateral retroperitoneal space, such as in other organs or in the contralateral retroperitoneal space.

### Immunohistochemistry and patient grouping

**Figure 1:**
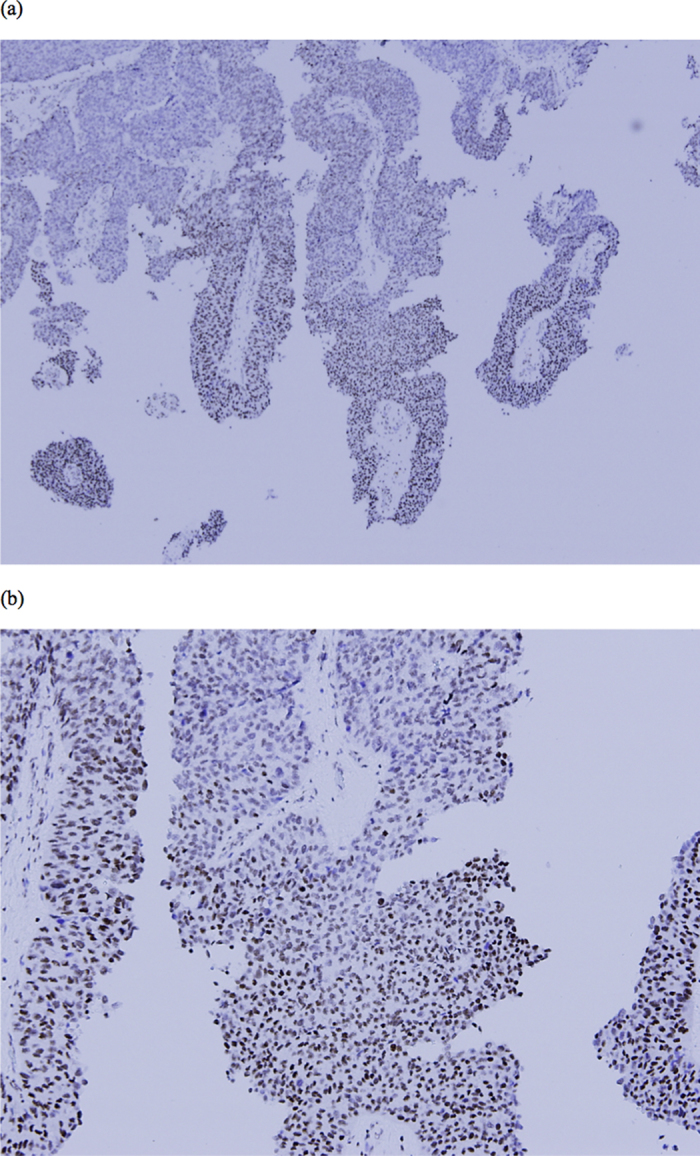
(**a**) 40X microscopic view of high ERβ expression specimen. (**b**) 100X microscopic view of high ERβ expression specimen.

We have tested the AR and ER-alpha in thirty randomized fifty T3 UTUC specimens initially. However, there is little AR and ER alpha expression in our series and this result might suggest that AR and ER-alpha may not occupy an important role in prediction of clinical prognosis in our series (Supplement file, Fig. 1). Therefore we choose ERβ as out study target. Immunostaining for estrogen receptor beta (ERβ) was performed on a fully automated Bond-Max system (Leica Microsystems). Slides carrying tissue sections cut from formalin-fixed, paraffin-embedded tissue microarray blocks were dried for 1 hour at 60 °C. These slides were then covered by Bond Universal Covertiles and placed into the Bond-Max instrument. All subsequent steps were performed automatically by the instrument according to the manufacturer’s instructions (Leica Microsystems). The following procedure was used: (1) deparaffinization of tissue on slides by rinsing with Bond Dewax Solution at 72 °C; (2) heat-induced epitope retrieval (antigen unmasking) with Bond Epitope Retrieval Solution 2 for 20 minutes at 100 °C; (3) peroxide block placement on the slides for 5 minutes at room temperature; (4) incubation with a mouse monoclonal anti-ERβ antibody at a dilution of 1:100 for 30 minutes at room temperature; (5) incubation with Post Primary reagent for 8 minutes at room temperature; (6) Bond Polymer placement on the slides for 8 minutes at room temperature; (7) color development with 3,3′-diaminobenzidine tetrahydrochloride (DAB) as a chromogen for 5 minutes at room temperature; and (8) hematoxylin counterstaining for 5 minutes. Slides were mounted and examined by light microscopy. Representative conventional tumor sections were selected to examine the pattern of ERβ presentation instead of tissue microarray (Fig. 1). Pathologist M.T.S., who was blinded to the oncologic outcome, scored the percentage of ERβ expression in UTUC representative slide and we choose 10% as the cut-off value. The percentage of ERβ expression more than 10% was thought to be high ERβ expression.

### Cell line study

**Figure 2:**
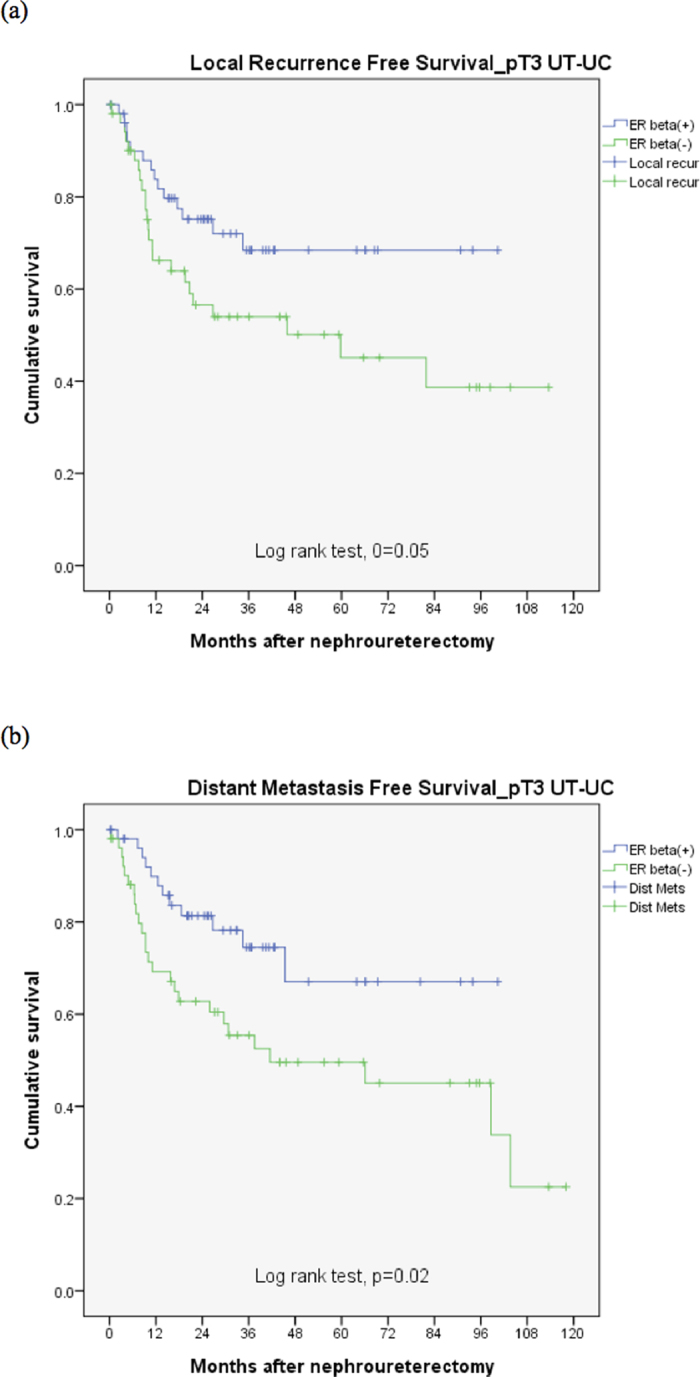
(**a**) ERβ effect on local recurrence free survival for pT3 UT-UC. (**b**) ERβ effect on distant metastasis free survival for pT3 UT-UC.

**Figure 3:**
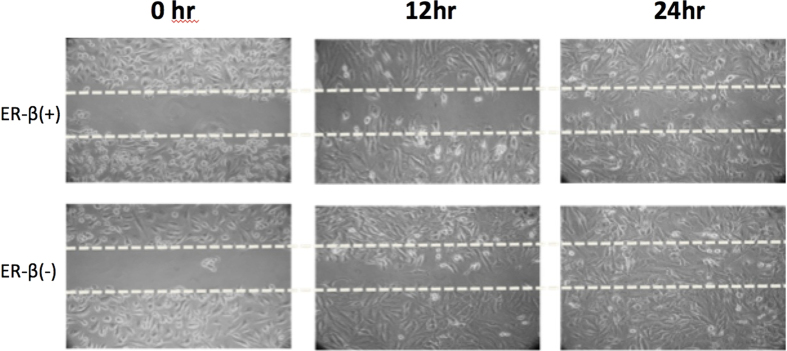
Wound healing assay revealed ERβ knock down UTUC cells were more proliferative in 24 hours observation.

We use BFTC-909 cell line (a cell line from renal pelvis patient), which is an ERβ expression cell line, for cancer behavioral analysis (Supplement file, Fig. 2). This cell line was cultured in Dulbecco’s modified Eagle’s medium, containing 10% heat-inactivated fetal bovine serum (FBS) at 37 °C in an atmosphere of 5% CO2. ERβ was knock down adequately by Origene ER-beta shRNA transfection (Supplement file, Fig. 3). Wound healing assay was performed to test the proliferation ability of cell line. Cell monolayers were then wounded by sterile pipette tips (1 ml) that generated a gap. Wounded monolayers were then washed three times with PBS to remove cell debris and incubated in 10% CD FBS medium for 24-hour observation. Migration trans-well assay was performed to assess the aggressiveness of cell line. In the upper layer of a cell permeable membrane and a solution containing the test agent is placed below the cell permeable membrane. Following an incubation period (37 °C, 5% CO2, 24 hours), the cells that have migrated through the membrane are stained and counted. We use DPN (diarylpropionitrile, 10 nM) as ERβ agonist and PHTPP (4-[2-phenyl-5,7-bis(trifluoromethyl)pyrazolo[1,5-a]- pyrimidin-3-yl]phenol, 150 nM) as ERβ antagonist. The cell migration number was counted after cisplatin(20 μM) treatment, DPN, and PHTPP for migration inhibition evaluation.

### Statistical analysis

SPSS version 17 software was used for all statistical analyses. The chi-square test and independent *t*-test were used for intergroup comparisons. The Kaplan-Meier method with log-rank test was used for time-to-event analysis. Multivariate Cox regression analysis was used to assess the independent roles of perioperative factors on systemic recurrence or cancer-specific death. A *p* value of ≤0.05 was defined to be statistically significant.

## Results

This retrospective study included samples collected between 2005 and 2012 from 105 patients with locally advanced UT-UC (pT3) and adequate quality of tissue for immunohistochemical stain, divided patients into ERβ-high (n = 52) or ERβ-low (n = 53) groups and then analyzed their oncologic outcomes. Table 1 reveals patient characteristics and all pathological features except infiltrating tumor architecture, which had a significantly higher incidence in the ERβ-low group (p = 0.004), were identical in both groups. Upper urinary tract urothelial carcinoma with low ERβ expression had worse local recurrence and distant metastasis-free survival compared with high ERβ expression (*p* = 0.05 and 0.02 respectively, Fig. 2). The results of multivariate analysis are listed in Table 2. All commonly known prognostic pathological factors were included for examination. Low ERβ expression was significantly correlated with local recurrence and distant metastasis in univariate analysis (*p* = 0.035 and 0.004 respectively) and multivariate analysis (*p* = 0.05 and 0.008 respectively).

**Figure 4:**
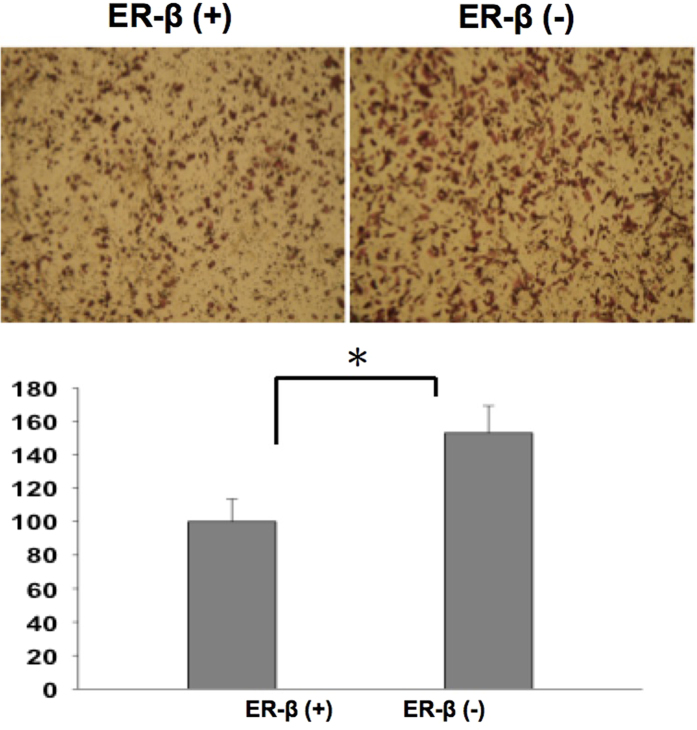
Migration assay revealed ERβ knock down UTUC cells were tend to more aggressive.

**Figure 5:**
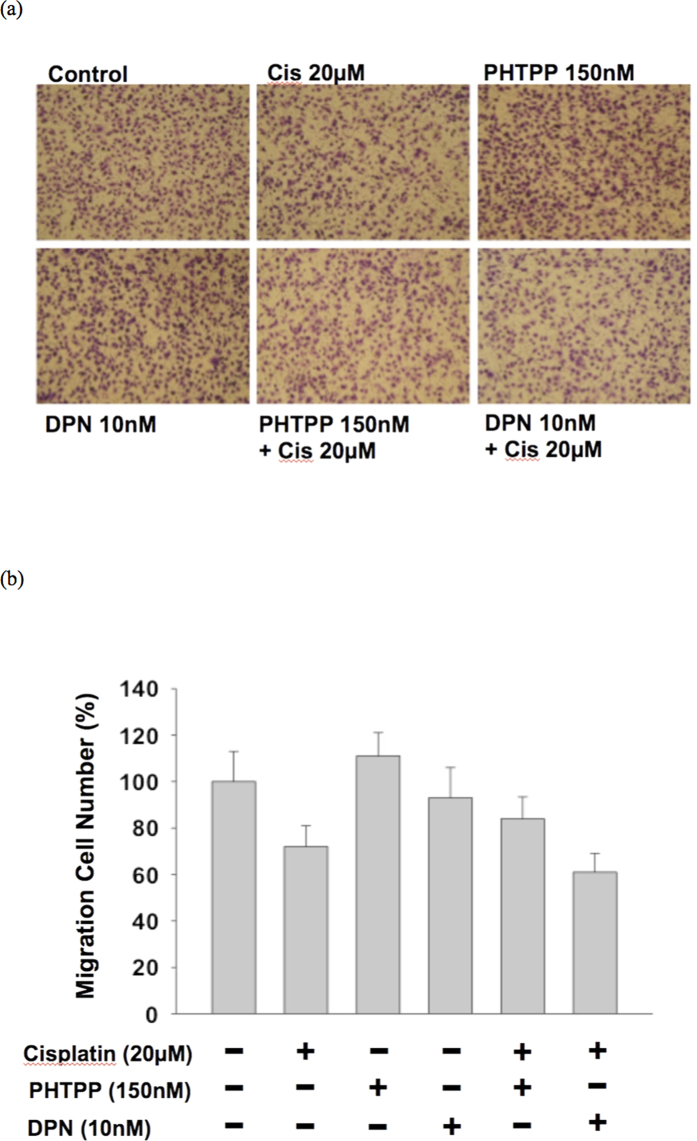
(**a**) The microscopic view for migration assessment of UTUC cell line by different combination of cisplatin, PHTPP, and DPN. (**b**) Cisplatin can cause less migration of UTUC cells. PHTPP causes UTUC cells more resistant to cisplatin treatment and DPN enhances the cisplatin effect.

The result of cell line validation showed that knock down of ERβ cause aggressive UTUC cancer cell proliferation behavior by wound healing assay (Fig. 3). The migration assay also revealed more aggressive UTUC cancer cell migration if ERβ was knocked down (Fig. 4). The cytotoxic and migration-inhibiting effect of ERβ on UTUC cell line was observed and compared by cisplatin treatment. ERβ agonist enhanced the cisplatin effect while ERβ antagonist cause UTUC cell more resistant to cisplatin treatment (Fig. 5).

## Discussion

UT-UC is rare and the treatment options are limited. The TNM staging for UT-UC is relatively simple and there are few markers for sub-classification of advanced stage UT-UC^11^. The current standard treatment for UT-UC is nephroureterectomy with bladder cuff excision. Much effort has been focused on prognostic pathological features or sub-classification of the current TNM staging system^3^,^4^,^5^. The subclassification of locally advanced UT-UC is a clinically important issue. Unlike localized UT-UC (pT0-2), patients with locally advanced stage pT3 UT-UC are thought to experience much higher disease recurrence, even after radical surgery. However, variant prognoses are still noted in pT3 UT-UC in clinical practice. Further sub-classification of such locally advanced stage disease will be helpful to identify patients with need of early adjuvant therapy. Therefore, patients with pT3 UT-UC were selected to identify markers predictive for oncologic outcome in this study.

The most common challenge of advanced UT-UC is the high prevalence of renal insufficiency. Ineligibility for cisplatin-based chemotherapy due to impaired renal function, especially after nephroureterectomy, leads to poor prognosis in such patients^12^. Several molecular markers have been proposed to be associated with oncologic outcomes and may be potential targets for treatment of UT-UC^13^,^14^,^15^,^16^. However, little has been reported about the independent prognostic role of markers in comparison with other aggressive pathological features. In this study, we selected common prognostic pathological features from a systemic review^10^ in order to identify if the expression of ERβ had an independent and predictive prognostic effect that might initiate further translational investigations.

Increasing evidence indicates that urothelial carcinoma is a potential endocrine-related malignancy^6^,^8^. The most well known examples of biomarkers in endocrine-related cancer are estrogen receptors in breast cancer, where individualized hormonal therapy has improved overall prognosis^17^. However, research on hormone receptors has focused mainly on UB-UC^6^,^8^. Though UB-UC and UT-UC have similar cellular origins, their cancer behaviors differ^18^. ERβ has been reported to be a potential target associated with urothelial carcinoma behavior^8^,^9^. To our knowledge, this is the first study focusing on the prognostic effect of ERβ on UT-UC.

The most common method for prognostic marker screening is tissue array. However, asymmetric distribution of ERβ was noted in our specimens. We think that a tissue array might lead to detection bias in the subsequent outcome analysis. In this study, formalin-fixed representative conventional tumor blocks were used to thoroughly examine ERβ expression. The urologist assessing the clinical oncological outcome was blinded to ERβ expression and the pathologist examining ERβ expression was blinded to the oncologic outcome. Our result revealed that ERβ independently predicts the oncologic outcome for pT3 UT-UC. Its prognostic role has been reported in several malignancies^8^,^19^,^20^,^21^. ERβ suppresses the cadherin switch and correlates with better oncologic outcome. It is thought that ERβ expression associates with better survival^8^. For non-organ-confined UT-UC, the cancer behavior is reasonably an important mechanism for local recurrence and even distant metastasis. In our cell line study, knock down of ERβ does cause aggressive UTUC cancer cell behavior. In addition, agonist activation of ERβ has been shown to be associated with cell sensitization to cisplatin cytotoxicity, which is compatible with our cell line study^21^. These reports further support our finding that positive ERβ expression is correlated with better prognosis.

The major limitation of this study is its retrospective design and we could only observe the natural course of the UT-UC behavior without any adjuvant or targeted treatment. A prospective study with ERβ and the response to adjuvant or targeted interventions may provide further useful clinical information. The advantages of this study were a careful examination of the prognostic role of ERβ compared with established prognostic factors by multivariate analysis and a focus on the oncologic outcome of advanced UT-UC (pT3). Multivariate analysis can eliminate the potential crossed bias from other significant pathological factors. In addition, a focused analysis on T3 UT-UC avoids the curative effect of radical surgery on organ-confined disease (pT0-2). In this study, ERβ still showed a significant association with oncologic prognosis. Further studies on adjuvant or targeted therapy on ERβ for UT-UC are deemed worthwhile.

## Conclusion

This study proposes that positive presentation of ERβ is correlated with better prognosis of pT3 UT-UC compared with commonly known aggressive pathological features. This result may help to identify patients requiring early adjuvant therapy or develop potential targeted therapies.

## Additional Information

**How to cite this article**: Luo, H. L. *et al*. Expression of Estrogen Receptor Beta Predicts Oncologic Outcome of pT3 Upper Urinary Tract Urothelial Carcinoma Better Than Aggressive Pathological Features. *Sci. Rep*. **6**, 24263; doi: 10.1038/srep24263 (2016).

## Supplementary Material

Supplementary Information

## Figures and Tables

**Table 1:** Patient characteristics.

	ER β (−)	ER β (+)	*p* value
No.	53	52	
Follow duration	39.5 ± 34.3	34.6 ± 23.9	0.404
Age	68.6 ± 10.8	68.6 ± 10.0	0.975
Smoking	7(13.2%)	7(13.5%)	0.969
Bladder cancer history	3(5.7%)	8(15.4%)	0.119
Infiltrating tumor	22(41.5%)	9(17.3%)	0.004
LN positive	2(3.8%)	4(7.7%)	0.414
LVI	27(50.9%)	20(38.5%)	0.144
CIS	16(30.2%)	20(38.5%)	0.452
SCC diff.	19(35.8%)	18(34.6%)	0.782
TN	17(32.1%)	27(51.9%)	0.060
Multifocal tumor	9(17.0%)	9(17.3%)	0.965
High grade	52(98.1%)	52(100%)	0.320
Bladder recurrence	9(17.0%)	11(21.2%)	0.653
Local recurrence	24(45.3%)	14(26.9%)	0.035
Distant metastasis	26(49.1%)	12(23.1%)	0.004
Cancer specific mortality	17(32.1%)	6(11.5%)	0.008

Abbreviation: TN = Tumor necrosis, CIS = Carcinoma *in situ*, LVI = Lymphovascular invasion, SCC diff = Squamous differentiation, LN = Lymph node.

**Table 2:** Multivariate analysis for prognostic factors about T3 UT-UC recurrence.

	Local recurrence		Distant metastasis	
	Univariate p value	Multivariate p value	Univariate p value	Multivariate p value
ER β Low vs High	0.035	0.05 HR = 2.6, 95%CI = 1.0~6.9	0.004	0.008 HR = 4.8, 95%CI = 1.5~15.4
Tumor type Infiltrating vs papillary	0.033	0.05 HR = 2.6, 95%CI = 1.0~7.9	0.092	
Nodal status Positive vs Negative	0.468		0.013	0.998
Lymphovascular invasion Present vs Absent	0.103		0.014	0.064
Carcinoma *in situ* Present vs Absent	0.399		0.399	
SCC differentiation Present vs Absent	0.554		0.868	
Tumor necrosis Present vs Absent	0.975		0.975	
Tumor multifocality Multiple vs Solitary	0.782		0.415	
Tumor grade High vs Low	0.182		0.449	
Gender Female vs Male	0.184		0.082	
Age > 70 vs <=70	0.301		0.531	
Smoking Yes vs No	0.524		0.080	
Previous bladder cancer Yes vs No	0.189		0.008	0.997
