# Supplementary material for: Expression of Estrogen Receptor Beta Predicts Oncologic Outcome of pT3 Upper Urinary Tract Urothelial Carcinoma Better Than Aggressive Pathological Features
Source: Sci Rep. 2016 Apr 7;6:24263. doi: 10.1038/srep24263 (PMC4823660; doi:10.1038/srep24263)

**Expression of Estrogen Receptor Beta Predicts Oncologic Outcome of pT3 Upper Urinary Tract Urothelial Carcinoma Better Than Aggressive Pathological Features**

Hao Lun Luo^a,b^, Ming Tse Sung^c^, Eing Mei Tsai^a,d,e^, Chang Shen Lin^a,f^, Nai Lun Lee^b^, Yueh-Hua Chung^b,g^, Po Hui Chiang^b^

a. Graduate Institute of Medicine, College of Medicine, Kaohsiung Medical University, Kaohsiung, Taiwan

b. Department of Urology, Kaohsiung Chang Gung Memorial Hospital and Chang Gung University College of Medicine, Kaohsiung, Taiwan

c. Department of Pathology, Kaohsiung Chang Gung Memorial Hospital and Chang Gung University College of Medicine, Kaohsiung, Taiwan

d. Department of Obstetrics and Gynecology, Kaohsiung Medical University Hospital, Kaohsiung Medical University, Kaohsiung, Taiwan

e. Center for Research Resources and Development, Kaohsiung Medical University, Kaohsiung City, Taiwan.

f. Department of Biological Sciences, National Sun Yat-sen University, Kaohsiung, Taiwan

g. Institute of Biomedical Sciences, National Sun Yat-sen University, Kaohsiung, Taiwan.

Hao Lun Luo and Ming Tse Sung contribute to this article equally

Corresponding author:

1. Professor Po Hui Chiang, Department of Urology, Kaohsiung Chang Gung Memorial Hospital, 123, Ta-Pei Road, Niaosung, Kaohsiung, Taiwan
2. Professor Eing Mei Tsai, Department of Obstetrics and Gynecology, Kaohsiung Medical University Hospital, Kaohsiung Medical University, Kaohsiung, Taiwan

Tel: +886-7-7317123 ext. 8094; Fax: +886-7-7317123 ext. 8004

E-mail: [tuoa480713@yahoo.com.tw](mailto:tuoa480713@yahoo.com.tw), [tsaieing@kmu.edu.tw](mailto:tsaieing@kmu.edu.tw)

Fig-1 The hormone receptors distribution in our UTUC database(Examination for randomized 30 pT3 UTUC specimens)


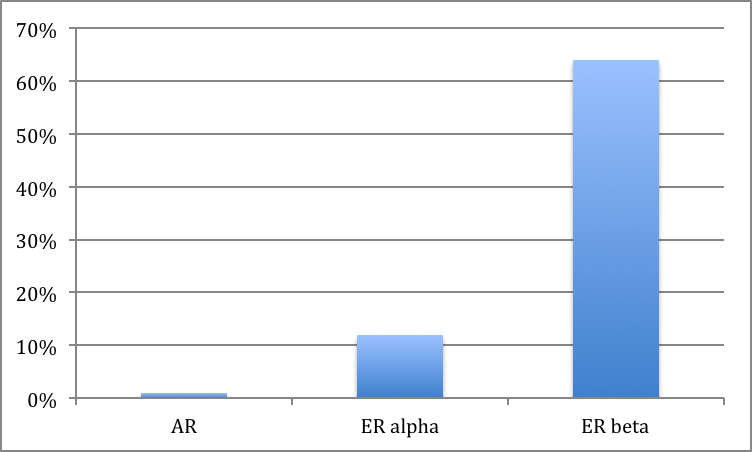


Fig-2

BFTC-909 is a ER beta positive cell line


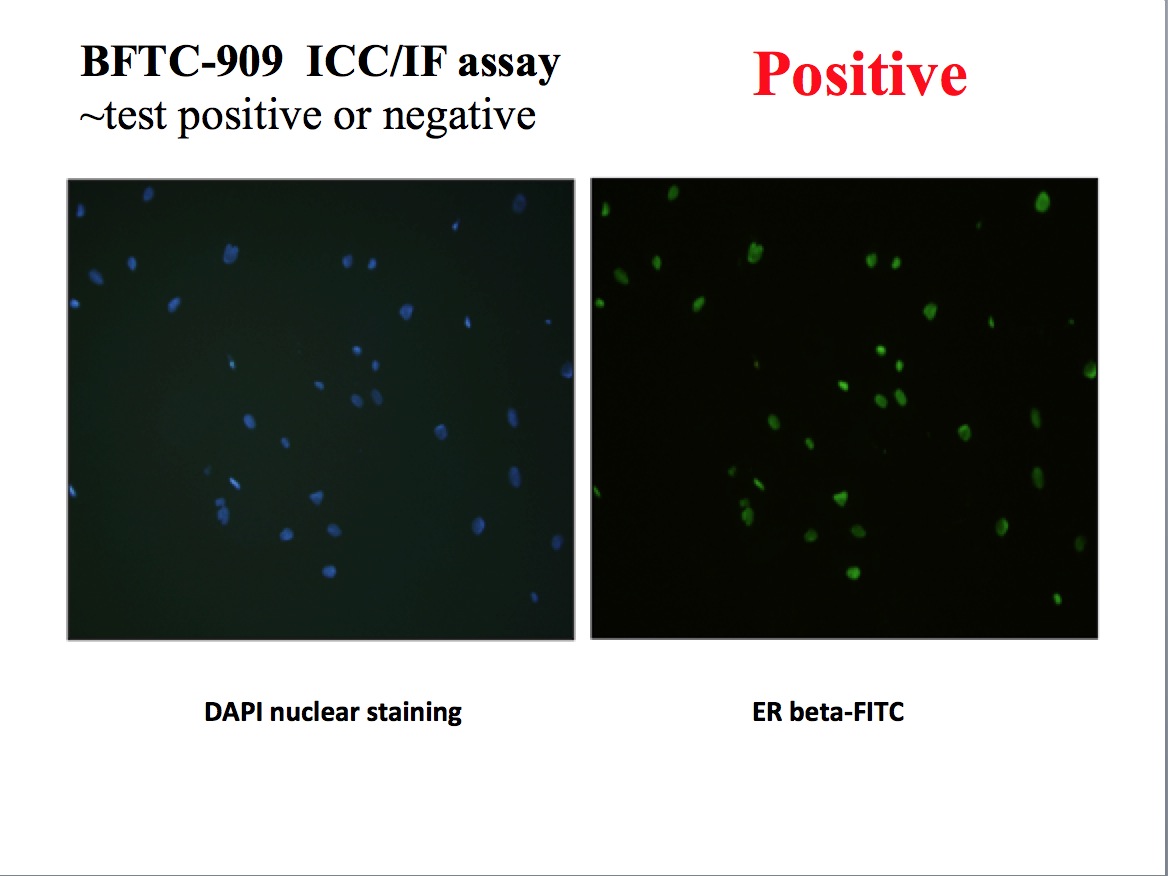


Fig 3


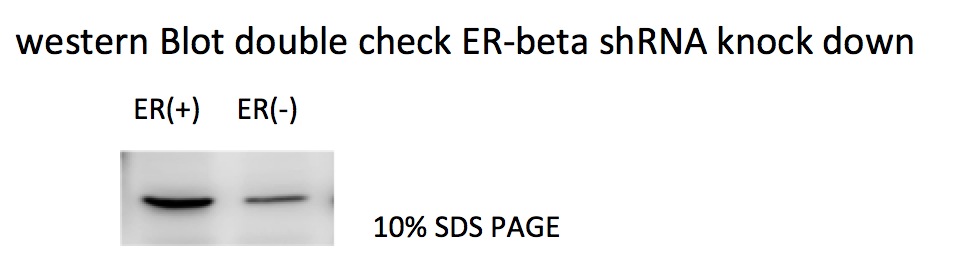


Fig-4


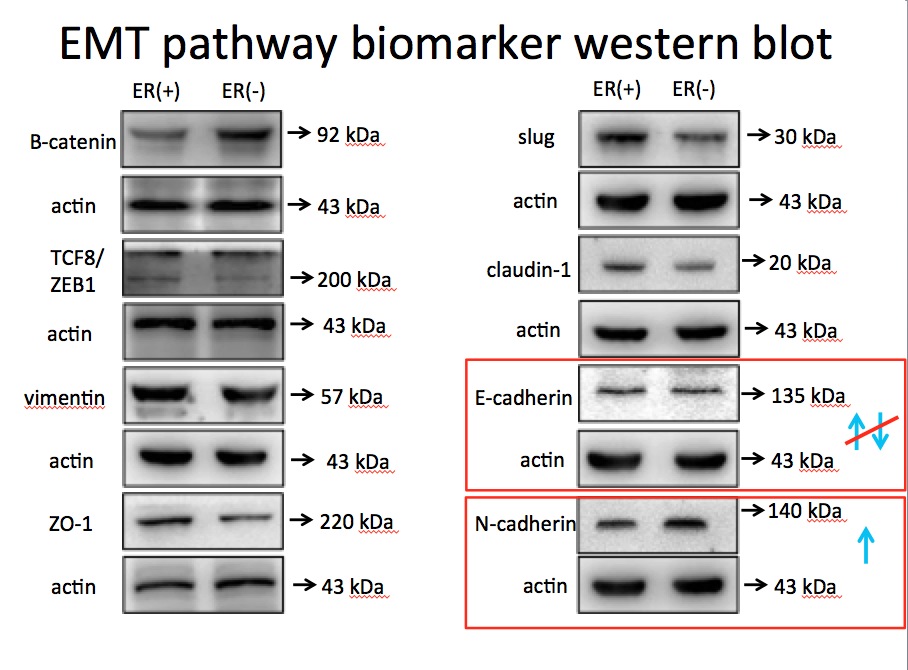

Supplement: Supplementary Information [file srep24263-s1.doc]
